# POSS Dental Nanocomposite Resin: Synthesis, Shrinkage, Double Bond Conversion, Hardness, and Resistance Properties

**DOI:** 10.3390/polym10040369

**Published:** 2018-03-26

**Authors:** Yizhi Liu, Xiaorong Wu, Yi Sun, Weili Xie

**Affiliations:** 1 Department of Astronautic Science and Mechanics, Harbin Institute of Technology, Harbin 150001, China; wxr@hit.edu.cn (X.W.); sunyi@hit.edu.cn (Y.S.); 2 Department of Stomatology, Harbin Medical University, Harbin 150001, China; xwl811@126.com

**Keywords:** dental resin, methacryl POSS, shrinkage, hardness, scratch resistance

## Abstract

Nanocomposite dental resins with 0, 2, 5, and 10 wt % methacryl polyhedral oligomeric silsesquioxane (POSS) as filler in the resin matrix were prepared by a light curing method.The atomic force microscopy (AFM), fourier transform infrared spectroscopy (FTIR), nanoindentation, and nanoscratch tests were carried out to study the effect of POSS contents on the compatibility, double bond conversion, volumetric shrinkage, hardness, modulus, and resistance of the dental resins. POSS was very uniformly dispersed and showed a good compatibility with the matrix. The double bond conversion increased and the volume reduced with the addition of POSS. As the POSS addition increased, the mechanical properties increased initially. Small addition of POSS remarkably enhanced the hardness and scratch resistance of the resin matrix.

## 1. Introduction

Increasingly, conventional alloys in the current dental field have been replaced by resin materials due to their advantages such as operation technique and aesthetic quality [1,2]. Nanofiller-reinforced dental composites show large advantages in large area restoration applications due to its easy processing and good biocompatibility [3,4]. In the past few years, the application of new nanofillers in dental resins to improve its mechanical property and anti-caries function has become a popular research topic [5,6,7,8,9].

**Figure 1:**
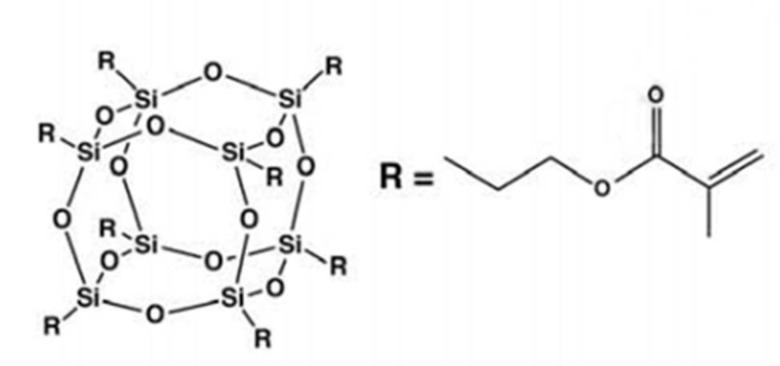
The molecule structure of methacryl polyhedral oligomeric silsesquioxane (POSS).

Hybrid organic-inorganic nanocomposites, which incorporate polyhedral oligomeric silsesquioxane (POSS) into polymeric matrices including dental resins, have received a considerable amount of attention. Polyhedral oligomeric silsesquioxane (POSS) is an organic-inorganic hybrid nanomaterial. Its formula is (RSiO_3/2_)_n_, where n is the number of the silicon atoms of the cage (1–3 nm in size) surrounded by organic corner groups R. There are very different types of POSS according to the various R organic groups [10,11].The physical mixture can be easily used to get together or react with monomer via copolymerization or blending to improve the physical properties [12,13,14]. Compared with traditional fillers, POSS can significantly improve the mechanical properties [15,16] of the composite and overcome the disadvantage of some polymer matrix materials, such as poor wear resistance, defects, and processing difficulties. At the same time, even the addition of a very small amount of POSS enhances the thermal [17,18,19,20], anti-oxygen erosion [21,22,23], and self-assembling [24,25,26,27] properties. Furthermore, the typical Si–O bonds and organic groups of POSS lead to evident improvements of the marginal adaptation of the matrix, ensuring good biocompatibility and comfort level [28], as well as remarkably reducing micro effusion risk and secondary caries [5,29]. In this study, colorless oil methacryl POSS (Figure 1) was chosen to be incorporated into neat resin due to its good compatibility.

Researchers have been making efforts to study the morphology, shrinkage, and physical properties of composite resins in the dental field and the aim is to focus on the synthesis of novel dental resin composites with low shrinkage and high mechanical properties. Hao Fong et al. [30] analyzed the following percentages (0, 2%, 5%, 10%, 25%, and 50%) of methacryl POSS (POSS-MA) in Bisphenol A glycerolate dimethacrylate (Bis-GMA), which work partially as novel dental restorative composites by the curing method. For the measurement of volumetric shrinkage, the curing time was set to be 1 min. After 1 hour post curing time, the light was turned on for another 30 s. His work indicated that Bis-GMA with POSS-MA did not affect the volumetric shrinkage, and the mechanical property of the composite was optimized only with a small percentage of POSS-MA substitution. Kleverlaan and Feilzer [31] evaluated the shrinkage, contraction stress, and tensile modulus of various commercially available dental resin composites. The shrinkage and contraction stress properties were enhanced by using pre-polymerized clusters. In our studies [32,33], novel dental nanocomposites with BG (barium oxide glass powder) and nano SiO_2_ fillers with different contents of methacryl POSS were developed, respectively. The polymerization shrinkage and mechanical properties were analyzed. To the best of our knowledge, there are not any reports about dental resin incorporated with the relatively low addition of POSS. But it seems that a low addition of POSS as nanofiller evidently enhances the performances of matrix [34,35].

In this study, dental resin composites with various amounts of POSS were processed using the light curing method. The influence of a low addition of POSS to dental resin was analyzed. The atomic force microscopy (AFM), FTIR, nanoindentation, and nanoscratch tests were performed, and the effects of the contents of POSS on the compatibility, double bond conversion, volumetric shrinkage, hardness, and resistance were studied.

## 2. Experimental

### 2.1. Materials

Bisphenol A glycerolate dimethacrylate (Bis-GMA) and Tri(ethylenglycol) dimethacrylate (TEGDMA) 98% were purchased from Aldrich Chemical Co. China (Shanghai, China). Camphorquinone (CQ, 97%) was used as a visible light photo-initiator and was selected with its co-initiator 2-(dimethylamino) ethylmethacrylate (DMAEMA, 98%) for this research. The nano filler, methacryl POSS, was purchased from Hybrid Plastics (Hattiesburg, MS, USA).

### 2.2. Preparation of POSS Composite Resins

A resin matrix solution containing Bis-GMA, TEGDMA, CQ, and DMAEMA was prepared by mixing in a light resistance environment after sufficient mixing. Then different percentages methacryl POSS, according to the quantities reported in Table 1, were added and blended uniformly by a magnetic stir bar.

The whole process should be performed under vacuum conditions. The mixtures went through a light curing process which lasted for 40 s at room temperature in stainless steel molds. After being soaked in distilled water at 37 °C for 24 h, they were placed in configured artificial saliva (according to ISOTR1021) at 36.5 °C for 4 more weeks. Finally, the dimensions of the specimens were measured accurately before testing.

## 3. Characterization

### 3.1. AFM Characterization

AFM topography images were obtained in Peak Force Tapping on the Bruker instruments (Bruker, Billerica, MA, USA). The scan rate was set to be 1 Hz and imaging was carried out on Multimode 8.

### 3.2. FTIR Characterization and Measurement of Double Bond Conversion

FTIR spectrometer (Avatar360, Nicolet, Madison, WI, USA) was used to carry out the Fourier transform infrared (FTIR) spectroscopy analysis. The scan range was from 4000 to 400 cm^−1^, with a resolution of 4 cm^−1^. 

The degree of double bond conversion (DC) was monitored by FTIR. The DC was calculated from the methacrylate C=C peak at 1636 cm^−1^ and normalized against the carbonyl C=O peak at 1720 cm^−1^ according to the Equation 1.

(1) $$DC(t)=\frac{{\left(\frac{A_{C=C}}{A_{C=O}}\right)}_{0}-{\left(\frac{A_{C=C}}{A_{C=O}}\right)}_{t}}{{\left(\frac{A_{C=C}}{A_{C=O}}\right)}_{0}}$$

where $A_{C=C}$ and $A_{C=O}$ are the absorbance peak areas at 1636 and 1720 cm^−1^, which are characteristic of methacrylate C=C and carbonyl, respectively. ${\left(\frac{A_{C=C}}{A_{C=O}}\right)}_{0}$ and ${\left(\frac{A_{C=C}}{A_{C=O}}\right)}_{t}$ were the initial and terminal ratio of these two absorbance peak areas.

### 3.3. Shrinkage

The densities of both uncured and cured resin samples were measured by a pycnometer to determine the polymerization shrinkage according to the Archimedes’ principle. The volumetric shrinkage was calculated using the following equation:

(2) ΔV%=(1−ρuncuredρcured)×100%

where $\rho_{uncured}$ and $\rho_{cured}$ are the density of uncured and cured resin specimens, respectively. They are calculated according to the following equations:

(3) $$\rho_{uncured}=\frac{m_{2}-m_{0}}{m_{1}-m_{0}}\times \rho_{w}$$

(4) $$\rho_{cured}=\frac{m_{s}}{m_{1}-m_{3}}\times \rho_{w}$$

where *m*_0_ is the mass of the empty bottle. Here, m_1_ is the mass of the bottle full of water at 20 °C; m_2_ is the mass of the bottle full of solution at 20 °C; m_3_ is the mass of the bottle with specimen and water; *m*_s_ is the mass of the specimen; and $\rho_{w}$ is the density of water.

### 3.4. Nanoindentation Testing

According to the Oliver-Pharr method [36,37], a nanoindentation test using G200 Nano Indenter (Agilent Technologies, Santa Clara, CA, USA) was used to evaluated the hardness of the nanocomposite resins. The tip of the diamond triangular pyramid Berkovich indenter (TB 20114 ISO) had a radius of about 20 nm. The maximum penetration depth during the indentation test was 2000 nm for each sample. A constant strain rate of 0.05 s^−1^ was ensured by the loading speed. The results of 8 samples were averaged to get the most accurate results.

### 3.5. Nanoscratch Testing

The resistance of the POSS-reinforced resin was evaluated by nanoscratch testing using a Nano Indenter G200 instrument (Agilent Technologies, USA). The method employed was the “G-Series single direction wear test”. The authors followed a test procedure similar as published elsewhere [38,39]. The same experimental environment and allowable temperature drift rate were used as in the nanoindentation test. The original topography and surface roughness of the sample were measured by pre-scanning the sample surface. The pre-scanning was carried out on 20% of the scratch length during the scratch test with a relatively light load of 50 μN. The load value during the scratch test was 200 mN and the load was applied on the indenter tip. The scratch length during the test was 400 μm and the speed was 10 μm/s. The scratch depth under the increasing scratch load could be obtained from the difference values by profiling the surface, and a total length of about 560 μm for each test was generated (including 80-μm rescanning and post-scanning at the two ends of the real scratch path), whereas the real effective scratch length was 400 μm, as applied to all specimens. Thus, the effective penetration depths, i.e., the scratch depth and the residual depth during and after the scratch test at different scratch distances, could be obtained. Three independent scratch tests were carried out for each sample and the results were averaged.

## 4. Results and Discussion

It was found during the tests that a higher percentage of the nano filler resulted in the deterioration of the transparency. However, this led to a high viscosity of the sample. Deterioration in the transparency thus led to a less light radiation fluence and a lower curing level of composites.

### 4.1. AFM

**Figure 2:**
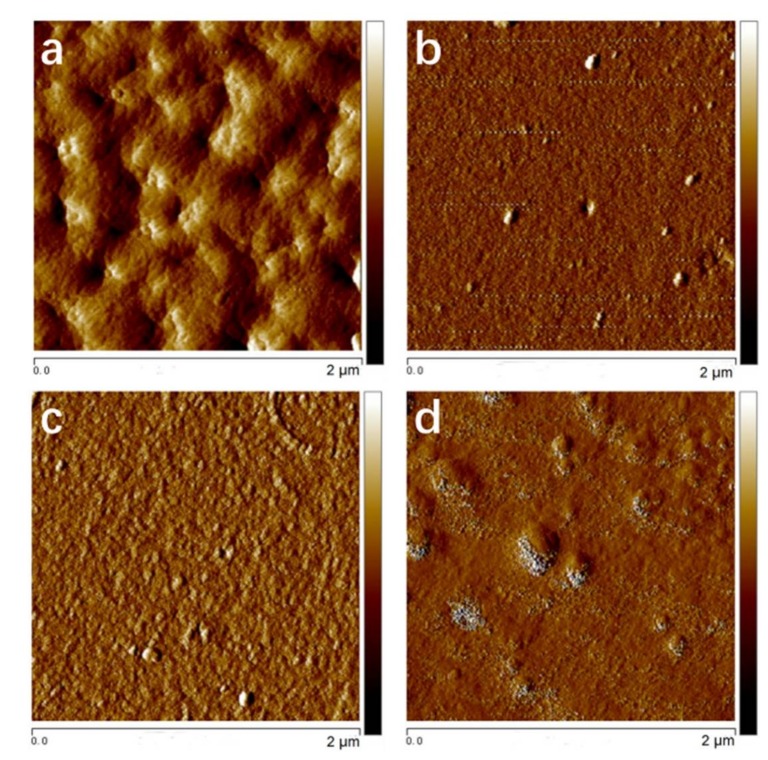
The atomic force microscopy (AFM) images of the structure of composite resins with different POSS additions: (**a**) P00, (**b**) P02, (**c**) P05, (**d**) P10.

The morphologies of a composite with the size of 2 μm × 2 μm, containing 0, 2, 5, and 10 wt % POSS, are shown in the AFM images in Figure 2, respectively. 

POSS showed a good compatibility with the matrix and could be uniformly dispersed at a low percentage of addition. However, once the POSS content reached 10 wt %, the aggregation of nanoparticles can be observed in the composite resin due to the self-assembly of POSS [40]. When the contents of POSS nanoparticles were low, the cavity and high surface roughness of the resin declined at the surface level, and the surface flatness and the porosity reduced. Due the viscous property of the POSS used in this work, as well as the long organic R chains around POSS which make the resin matrix molecules to distribute uniformly, the surfaces of all POSS composite resins are smoother than the one of the neat resin at the nano-size level in roughness. The low addition of POSS improves the surface smoothness.

### 4.2. FTIR Analysis and Double Bond Conversion (DC)

**Figure 3:**
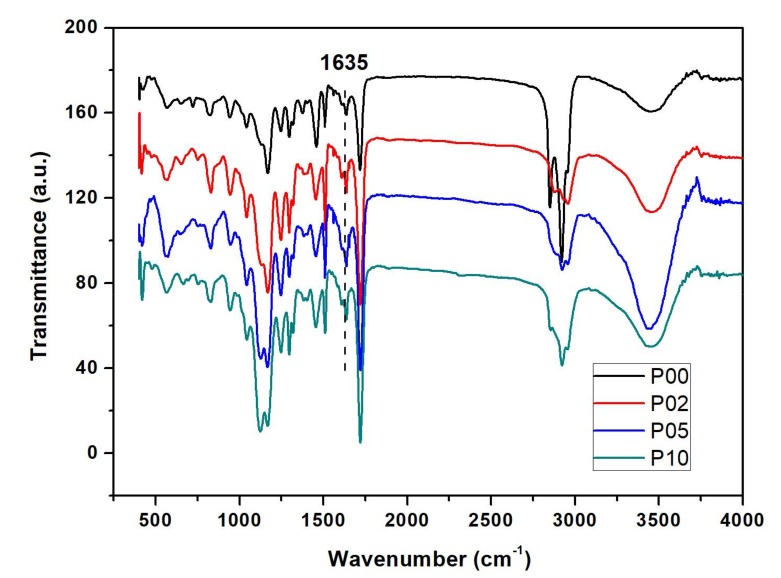
FTIR spectra of composite resins with different POSS additions.

The FTIR spectra of composite resins with different POSS contents after curing are shown in Figure 3. The band at 1635 cm^−1^ was attributed to the characteristic absorption peaks of the C=C bond. When the curing happened, the methacrylate double bonds partly converted and the resins polymerized [41]. The intensity of C=C absorption at 1635 cm^−1^ became evidently weaker.

**Figure 4:**
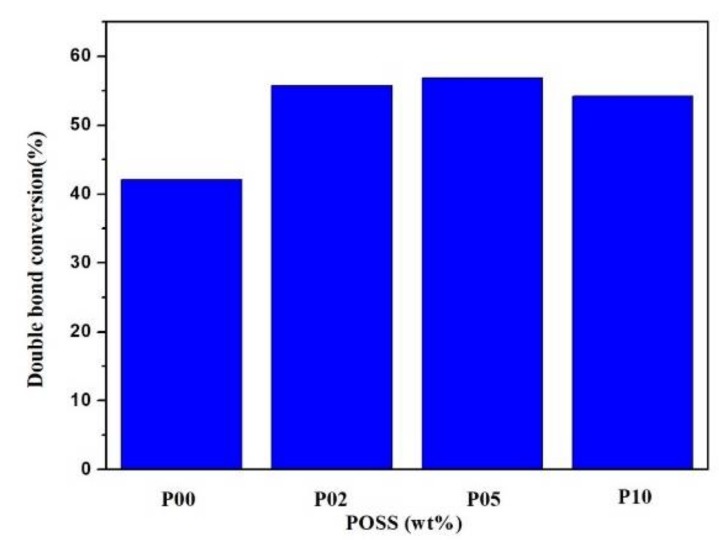
Double bond conversion of composite resins with different POSS additions.

The values of double bond conversion of composite resins with different POSS contents are shown in Figure 4. In this study, the curing process at room temperature took 40 s for each sample. The double bond conversion of the resin matrix was 42.1%. The double bond conversion of the POSS nanocomposite resin reached up to 55%. The variation trend is not obvious with the increasing addition even to 10 wt %. It can be explained by the fact that the uniformly distributed POSS in the polymer matrix at the nano-level allows the resin matrix to react more easily, which obviously increased the curing effect [42].

### 4.3. Volumetric Shrinkage

In this subsection, the shrinkage of composite resins synthesized were studied. The value of volumetric shrinkage of the resin matrix was 10.65%. As the percentage of POSS increased, the volume shrinkage of composites became lower. The values were about 9.07%, 9.85%, and 7.81% for the additions of 2%, 5%, and 10%, respectively.

In our previous work [43], the densities of uncured and cured models were calculated and the volume shrinkages of resins with different weight percentages of POSS were estimated. Compared with the results of the previous work, the values were almost the same. The results from other studies in the literatures also showed that the shrinkage values of typical resins of dental methacrylate-based monomers were between 6% and 10% [31,44,45]. Before the curing process, large chain monomers such as Bis-GMA and TEGDMA were loosely dispersed in the matrix. After curing, the reaction with monomers occurred. The movement of chains was not combined by amounts of cross-linked points and the free volume of resins changed remarkably. This results in a remarkable decrease of the shrinkage of resins. POSS has a three-dimensional organic-inorganic cage structure and its volume is much larger than those of Bis-GMA and TEGDMA. When POSS is incorporated in resins, it can attract and even entwine monomer chains. The monomers can disperse more tightly. POSS limits the change of the free volume of matrix during the reaction and the volume of the nanocage of POSS itself does not change after the reaction. In brief, the volume shrinkage of POSS dental composites reduces.

Besides, our previous experimental research and the study of Fong also investigated the volume shrinkage of the composite resins with an additional 60 and 70 wt % of BG powder [30,33]. The values of the shrinkage of POSS composites resin with a large percentage of additional BG powder were lower than the results reported in this work (without inorganic powder). The reasons are because the incorporation of a percentage of inorganic filler, BG, was very high and the volume of it did not change after the reaction. Consequently, the volumetric shrinkage of resins was very low. Even though, the trend was similar with the results of our previous research.

### 4.4. Hardness and Elastic Modulus

The hardness and resistance are two of the most important properties for dental restorations. In this study, the resistance of the POSS dental composites was evaluated by the scratch depth. The scratch depth can be used to evaluate the deformation resistance and hardness of the samples at a specific load [46,47]. The hardness, elastic modulus, and scratch depth of the nanocomposite resin were obtained by nanotesting technology. It should be emphasized that the sample was made of organic resin without traditional inorganic filler. Before the testing, the samples were placed in the configured artificial saliva at 36.5 °C for 4 weeks.

**Figure 5:**
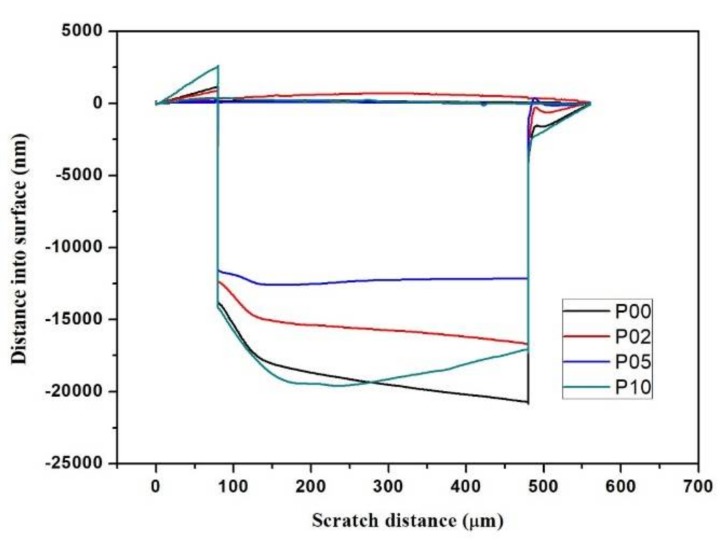
Influence of composite resins with different POSS additions on the scratch profiles.

The testing results of the hardness, elastic modulus, and the average scratch depth are listed in Table 2. The influence of composite resins with different POSS contents on the scratch profiles is shown in Figure 5. A total length of about 560 μm for each test was generated (including 80-μm pre-scanning and post-scanning at the two ends of real scratch path). The scratch region was from 80 to 480 μm in the scratch distance, in which the profile was essentially affected by the load value, hardness, and elasticity, revealing the scratch resistance property of a sample. 

As the POSS addition increased, the mechanical properties increased initially. Once it exceeded 5 wt %, the mechanical properties fell down. A small addition of POSS remarkably enhanced the hardness and the scratch resistance of the resin matrix. These properties showed the similar variation trend according to the percentage of POSS, which indicated that it had the same influence on the composites’ matrix. POSS, serving as a nucleating agent, attracts matrix chains, which increases the rigidity of the systems and enhances the mechanical properties. While a larger content of POSS perhaps results in a large reduction of the crosslinks among the matrix chains owing to the aggregation of POSS, it enables the polymer matrix to form plastic flow easily and reduces the mechanical properties [12,42,48]. The novel dental composite with 5 wt % POSS addition possesses superior performances in the relative increments of hardness, elastic modulus, and scratch depth.

Compared with SiO_2_, POSS is a unique kind of filler with a hard inorganic Si–O cage core surrounded by soft organic chains. The overall performance of POSS derives from the combined action of hard “core” and soft “shell” [49]. When the addition is small, POSS, as a nucleating agent, attracts polymer chains and forms more condensed particles. POSS can increase the bonding energy of the resin matrix and enhance the mechanical properties [50,51]. If the composites have good dispersion, the strong stress transfer is efficient [52,53]. The interface adhesion of the matrix and the filler is strengthened and most loads are carried by the hard core of POSS; as a result, the mechanical properties increase. When the addition of the filler is high, on one hand, large aggregation occurs. On the other hand, the distance between the filler is reduced and the stress transfer at the filler and matrix interface is inefficient. The R group of POSS twines together and the constraint level enhances, which results in an increasing thickness of the soft shell. The ability of the hard inorganic Si–O cage to act as an enhanced agent reduces, and the one of soft organic R groups increases [54,55]. 

Compared with the values of the hardness in our previous work [33], it is found the hardness values achieved in the current research were lower. This is because that some kinds of inorganic fillers are not added into the dental composite resins at high contents. In clinical applications, we find that although the hardness increases with the high addition of inorganic filler, some other key properties of dental resins, such compressive strength, become low after molding. As a result, the bulk of composite resins generates cracks easily and portions of it eventually turn into small particles due to frequent chewing. It can produce some problems in clinical applications when a large addition inorganic filler with a big particle size is employed. Certainly, the physical properties of dental products need to be as close as possible to those of dentin. So, it seems an excellent choice to base the preparation of dental restoration materials on organic resins only. Such organic resins derived from monomers can be prepared by polymerization or incorporated with only a small addition of inorganic filler.

## 5. Conclusions

In this paper, POSS is shown to be very compatible with the matrix. Weight fractions of 2%, 5%, and 10% of POSS in the resin matrix lead to an increase of the double bond conversion to about 55% when the curing time of each sample is 40 s. The values of volumetric shrinkage are about 9.07%, 9.85%, and 7.81%, respectively, at the above weight fractions. POSS limits the change in the free volume of the matrix during the reaction, but the volume of the nanocage of POSS itself does not change after the reaction. Thus, the shrinkage of resins with incorporated POSS decreases.

As the content of POSS increases, the mechanical properties increase initially. The addition of a small amount of POSS remarkably enhances the hardness and the scratch resistance of the resin matrix. Once it exceeds 5 wt %, the mechanical properties deteriorate. POSS, serving as a nucleating agent, attracts matrix chains and thus increases the rigidity of the systems and enhances the mechanical properties. However, a larger content of POSS may result in a large reduction of the crosslinks among matrix chains owing to the aggregation of POSS.

## Figures and Tables

**Table 1:** The compositions proportion of POSS hybrid dental nanocomposites.

	Resin Matrix				Nanofiller
	Resin System		Light Photo-Initiator		
	Bis-GMA	TEGDMA	CQ	DMAEMA	methacryl POSS
P00	49.5	49.5	0.5	0.5	0
P02	47.5	49.5	0.5	0.5	2
P05	44.5	49.5	0.5	0.5	5
P10	39.5	49.5	0.5	0.5	10

**Table 2:** The characterization of the hardness, elastic modulus, and average scratch depth of samples.

Samples	Hardness (GPa)	Elastic Modulus (GPa)	Average Scratch Depth (nm)
P00	0.204 ± 0.008	2.931 ± 0.163	−19,111 ± 502
P02	0.243 ± 0.013	3.388 ± 0.213	−15,684 ± 556
P05	0.322 ± 0.019	4.136 ± 0.204	−12,349 ± 473
P10	0.285 ± 0.017	3.491 ± 0.241	−18,331 ± 698

## References

[1] Buonocore M.G. (1955). A simple method of increasing the adhesion of acrylic filling materials to enamel surfaces. J. Dent. Res..

[2] Van Landuyt K.L., Snauwaert J., De Munck J., Peumans M., Yoshida Y., Poitevin A., Coutinho E., Suzuki K., Lambrechts P., Van Meerbeek B. (2007). Systematic review of the chemical composition of contemporary dental adhesives. Biomaterials.

[3] Van Nieuwenhuysen J.P., D’Hoore W., Carvalho J., Qvist V. (2003). Long-term evaluation of extensive restorations in permanent teeth. J. Dent..

[4] Miao X., Li Y., Zhang Q., Zhu M., Wang H. (2012). Low shrinkage light curable dental nanocomposites using SiO2 microspheres as fillers. Mater. Sci. Eng. C.

[5] Yudovin-Farber I., Beyth N., Nyska A., Weiss E.I., Golenser J., Domb A.J. (2008). Surface characterization and biocompatibility of restorative resin containing nanoparticles. Biomacromolecules.

[6] Liu Y., Tan Y., Lei T., Xiang Q., Han Y., Huang B. (2009). Effect of porous glass-ceramic fillers on mechanical properties of light-cured dental resin composites. Dent. Mater..

[7] Moharamzadeh K., Van Noort R., Brook I., Scutt A. (2007). HPLC analysis of components released from dental composites with different resin compositions using different extraction media. J. Mater. Sci..

[8] Xu H.H., Moreau J.L., Sun L., Chow L.C. (2008). Strength and fluoride release characteristics of a calcium fluoride based dental nanocomposite. Biomaterials.

[9] Tanimoto Y., Nemoto K. (2004). Influence of particle size of fillers on frictional wear of dental composite resins. Compos. Interfaces.

[10] Blanco I., Abate L., Bottino F.A., Bottino P. (2014). Synthesis, characterization and thermal stability of new dumbbell-shaped isobutyl-substituted POSSs linked by aromatic bridges. J. Therm. Anal. Calorim..

[11] Blanco I., Bottino F., Abate L. (2016). Influence of n-alkyl substituents on the thermal behaviour of Polyhedral Oligomeric Silsesquioxanes (POSSs) with different cage’s periphery. Thermochim. Acta.

[12] Liu Y., Sun Y., Zeng F., Liu J., Ge J. (2013). Effect of POSS nanofiller on structure, thermal and mechanical properties of PVDF matrix. J. Nanopart. Res..

[13] Ayandele E., Sarker B., Alexandridis P. (2012). Polyhedral Oligomeric Silsesquioxane (POSS)-Containing Polymer Nanocomposites. Nanomaterials.

[14] Blanco I., Bottino F.A., Cicala G., Latteri A., Recca A. (2014). Synthesis and characterization of differently substituted phenyl hepta isobutyl-polyhedral oligomeric silsesquioxane/polystyrene nanocomposites. Polym. Compos..

[15] Dodiuk-Kenig H., Maoz Y., Lizenboim K., Eppelbaum I., Zalsman B., Kenig S. (2006). The effect of grafted caged silica (polyhedral oligomeric silesquioxanes) on the properties of dental composites and adhesives. J. Adhes. Sci. Technol..

[16] Liu Y., Sun Y., Zeng F., Chen Y., Li Q., Yu B., Liu W. (2012). Morphology, crystallization, thermal, and mechanical properties of poly(vinylidene fluoride) films filled with different concentrations of polyhedral oligomeric silsesquioxane. Polym. Eng. Sci..

[17] Xu Z., Zhao Y., Wang X., Lin T. (2013). A thermally healable polyhedral oligomeric silsesquioxane (POSS) nanocomposite based on Diels-Alder chemistry. Chem. Commun..

[18] Huang Y., Gong S.-D., Huang R., Cao H.-J., Lin Y.-H., Yang M., Li X. (2015). Polyhedral oligomeric silsesquioxane containing gel polymer electrolyte based on a PMMA matrix. RSC Advances.

[19] Fina A., Tabuani D., Carniato F., Frache A., Boccaleri E., Camino G. (2006). Polyhedral oligomeric silsesquioxanes (POSS) thermal degradation. Thermochim. Acta.

[20] Blanco I., Bottino F.A. (2012). Effect of the substituents on the thermal stability of hepta cyclopentyl, phenyl substitued-Polyhedral oligomeric silsesquioxane (hcp-POSS)/polystyrene (PS) nanocomposites. AIP Conf. Proc..

[21] Minton T.K., Wright M.E., Tomczak S.J., Marquez S.A., Shen L., Brunsvold A.L., Cooper R., Zhang J., Vij V., Guenthner A.J. (2011). Atomic Oxygen Effects on POSS Polyimides in Low Earth Orbit. ACS Appl. Mater. Interfaces.

[22] Liu Y.Z., Sun Y., Zeng F.L., Zhang Q.H., Geng L. (2014). Characterization and analysis on atomic oxygen resistance of POSS/PVDF composites. Appl. Surf. Sci..

[23] Zeng F., Peng C., Liu Y., Qu J. (2015). Reactive Molecular Dynamics Simulations on the Disintegration of PVDF, FP-POSS and Their Composite during Atomic Oxygen Impact. J. Phys. Chem. A.

[24] Wu Y.C., Kuo S.W. (2012). Self-assembly supramolecular structure through complementary multiple hydrogen bonding of heteronucleobase-multifunctionalized polyhedral oligomeric silsesquioxane (POSS) complexes. J. Mater. Chem..

[25] Lin Y.C., Kuo S.W. (2012). Hierarchical self-assembly structures of POSS-containing polypeptide block copolymers synthesized using a combination of ATRP, ROP and click chemistry. Polym. Chem..

[26] Kuo S.W., Chang F.C. (2011). POSS related polymer nanocomposites. Prog. Polym. Sci..

[27] Blanco I., Bottino F.A., Cicala G., Cozzo G., Latteri A., Recca A. (2015). Synthesis and thermal characterization of new dumbbell shaped POSS/PS nanocomposites: Influence of the symmetrical structure of the nanoparticles on the dispersion/aggregation in the polymer matrix. Polym. Compos..

[28] Siang Soh M., Sellinger A., Uj Yap A. (2006). Dental nanocomposites. Curr. Nanosci..

[29] Eick J.D., Smith R.E., Pinzino C.S. (2006). Stability of silorane dental monomers in aqueous systems. J. Dent..

[30] Fong H., Dickens S.H., Flaim G.M. (2005). Evaluation of dental restorative composites containing polyhedral oligomeric silsesquioxane methacrylate. Dent. Mater..

[31] Kleverlaan C.J., Feilzer A.J. (2005). Polymerization shrinkage and contraction stress of dental resin composites. Dent. Mater..

[32] Liu Y., Sun Y., Zeng F., Xie W., Liu Y., Geng L. (2014). Effect of nano SiO_2_ particles on the morphology and mechanical properties of POSS nanocomposite dental resins. J. Nanopart. Res..

[33] Wu X., Sun Y., Xie W., Liu Y., Song X. (2010). Development of novel dental nanocomposites reinforced with polyhedral oligomeric silsesquioxane (POSS). Dent. Mater..

[34] Markovic E., Clarke S., Matisons J., Simon G.P. (2008). Synthesis of POSS-methyl methacrylate-based cross-linked hybrid materials. Macromolecules.

[35] Sanchez-Soto M., Schiraldi D.A., Illescas S. (2009). Study of the morphology and properties of melt-mixed polycarbonate-POSS nanocomposites. Eur. Polym. J..

[36] Oliver W.C., Pharr G.M. (1992). An Improved technique for determining hardness and elastic modulus using load and displacement sensing indentation experiments. J. Mater. Res..

[37] Oliver W.C., Pharr G.M. (2004). Measurement of hardness and elastic modulus by instrumented indentation: Advances in understanding and refinements to methodology. J. Mater. Res..

[38] Xingwen Z., Lijiang H., Dezhi S. (2006). Nanoindentation and nanoscratch profiles of hybrid films based on (γ-methacrylpropyl)trimethoxysilane and tetraethoxysilane. Acta Mater..

[39] Zeng F., Liu Y., Sun Y., Hu E., Zhou Y. (2012). Nanoindentation, nanoscratch, and nanotensile testing of poly(vinylidene fluoride)-polyhedral oligomeric silsesquioxane nanocomposites. J. Polym. Sci. Part B Polym. Phys..

[40] Blanco I., Bottino F.A., Cicala G., Latteri A., Recca A. (2013). A kinetic study of the thermal and thermal oxidative degradations of new bridged POSS/PS nanocomposites. Polym. Degrad. Stab..

[41] Stansbury J., Dickens S.H. (2001). Determination of double bond conversion in dental resins by near infrared spectroscopy. Dent. Mater..

[42] Schiraldi D.A., Iyer S. (2007). Role of specific interactions and solubility in the reinforcement of bisphenol A polymers with polyhedral oligomeric silsesquioxanes. Macromolecules.

[43] Song X., Sun Y., Wu X., Zeng F. (2011). Molecular dynamics simulation of a novel kind of polymer composite incorporated with polyhedral oligomeric silsesquioxane (POSS). Comput. Mater. Sci..

[44] Dupasquier F., Gritsch K., Farlay D., Zydowicz N., Grosgogeat B. (2007). Mechanical properties and polymerization shrinkage of dental nanohybrid composites. Eur. Cells Mater..

[45] Chen M.H., Chen C.R., Hsu S.H., Sun S.P., Su W.F. (2006). Low shrinkage light curable nanocomposite for dental restorative material. Dent. Mater..

[46] Sanchez J., El-Mansy S., Sun B., Scherban T., Fang N., Pantuso D., Ford W., Elizalde M., Martınez-Esnaola J., Martın-Meizoso A. (1999). Cross Sectional nanoindentation: A new technique for thin film interfacial adhesion characterization. Acta Mater..

[47] Kim B., Ko M. (2009). The assessment of the fracture behavior in spin-on organosilicates by nanoindentation and nanoscratch tests. Thin Solid Films.

[48] Liu Y., Sun Y., Yu J., Zeng F., Xiao Y., Geng L., Lv P. (2016). Study on different behaviour of poly(vinylidene fluoride)-polyhedral oligomeric silsesquioxane nanocomposites in mechanical properties and nano-tensile testing. Mater. Res. Innov..

[49] Baldi F., Bignotti F., Fina A., Tabuani D., Riccò T. (2007). Mechanical characterization of polyhedral oligomeric silsesquioxane/polypropylene blends. J. Appl. Polym. Sci..

[50] Martins J.N., Bassani T.S., Oliveira R.V.B. (2012). Morphological, viscoelastic and thermal properties of poly(vinylidene Fluoride)/POSS nanocomposites. Mater. Sci. Eng. C.

[51] Liu L., Tian M., Zhang W., Zhang L., Mark J.E. (2007). Crystallization and morphology study of polyhedral oligomeric silsesquioxane (POSS)/polysiloxane elastomer composites prepared by melt blending. Polymer.

[52] Schadler L., Giannaris S.a., Ajayan P. (1998). Load transfer in carbon nanotube epoxy composites. Appl. Phys. Lett..

[53] Gojny F., Wichmann M., Köpke U., Fiedler B., Schulte K. (2004). Carbon nanotube-reinforced epoxy-composites: Enhanced stiffness and fracture toughness at low nanotube content. Compos. Sci. Technol..

[54] Kopesky E.T., McKinley G.H., Cohen R.E. (2006). Toughened poly(methyl methacrylate) nanocomposites by incorporating polyhedral oligomeric silsesquioxanes. Polymer.

[55] Špírková M., Duchek P., Strachota A., Poręba R., Kotek J., Baldrian J., Šlouf M. (2011). The role of organic modification of layered nanosilicates on mechanical and surface properties of organic-inorganic coatings. J. Coat. Technol. Res..

